# Memorable first impressions

**DOI:** 10.7554/eLife.98274

**Published:** 2024-05-03

**Authors:** Emilio Salinas, Bashirul I Sheikh

**Affiliations:** 1 Department of Translational Neuroscience, Wake Forest University School of Medicine Winston-Salem United States; 2 Neuroscience Graduate Program, Wake Forest University School of Medicine Winston-Salem United States

**Keywords:** short-term memory, population coding, temporal dynamics, delay encoding, iconic memory, Human

## Abstract

Our ability to recall details from a remembered image depends on a single mechanism that is engaged from the very moment the image disappears from view.

**Related research article** Tomić I, Bays PM. 2023. A dynamic neural resource model bridges sensory and working memory. *eLife* **12**:RP91034. doi: 10.7554/eLife.91034.

Look out the window and see what stands out. Perhaps you notice some red and pink azaleas in full bloom. Now close your eyes and picture that scene in your mind. Initially, the colors and silhouettes linger vividly, but the details wither rapidly, leaving only a faded version of the image. As time passes, the accuracy with which an image can be recalled drops abruptly.

Memory is a critical, wonderful, multifaceted mental capacity that relies on many structures and mechanisms throughout the brain (Baddeley, 2003; Squire and Wixted, 2011; Schacter et al., 2012). This is not surprising, given the diversity of timescales and data types – such as images, words, facts and motor skills – that we can remember. Studies have shown that our visual memories are strongest immediately after an image disappears, remaining reliable for about half a second. This has traditionally been attributed to ‘iconic’ memory, which is thought to rely on a direct readout of stimulus-driven activity in visual circuits in the brain. In this case, the memory remains vivid because, after the stimulus (i.e., the image) has been removed, the visual activity takes some time to decay (Sperling, 1960; Pratte, 2018; Teeuwen et al., 2021).

In contrast, recalling an image a second or so after it has disappeared engages a different type of memory – visual working memory – that relies on information stored in different circuits in the frontal lobe (Pasternak and Greenlee, 2005; D’Esposito and Postle, 2015). Although not as vivid, the stored image remains stable for much longer. This is because, despite being more robust, the storage capacity for visual working memory is more limited: fewer items and less detail can be recalled from a remembered image. Together, these findings led to the idea that there are two distinct short-term memory mechanisms. Now, in eLife, Ivan Tomić and Paul Bays report strong evidence indicating that iconic memory and visual working memory are part of the same recall mechanism (Tomić and Bays, 2023).

Tomić and Bays – who are based at the University of Zagreb and the University of Cambridge – first constructed a detailed computational model to describe how sensory information is passed to a visual working memory circuit for storage and later recall (Figure 1). In this model, visual neurons respond to the presentation of an image containing a few items. This stimulus causes sensory activity to rise smoothly while the input lasts, and to decay once the stimulus ceases, consistent with previous experiments (Teeuwen et al., 2021). This sensory response then drives a population of visual working memory neurons that can sustain their activity in the absence of a stimulus, although this activity will eventually be corrupted due to noise (Wimmer et al., 2014; DePasquale et al., 2018). An important feature of the model is that each remembered item is allocated an equal fraction of the maximum possible working memory activity.

**Figure 1.**
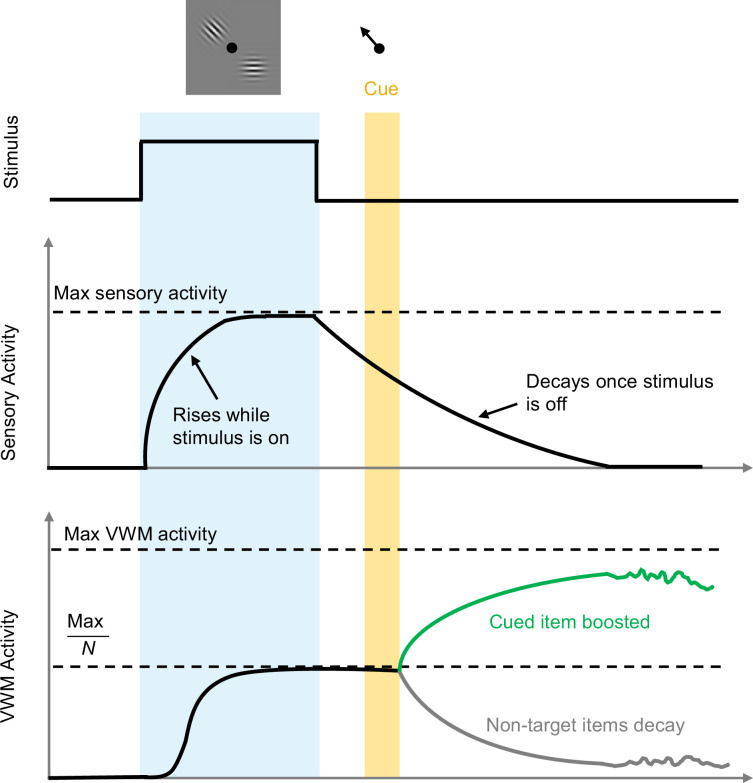
Timeline of events during stimulus presentation and storage. A visual stimulus (grey box containing pattern) with *N* items is presented for a period of time (pale blue region). Sensory activity increases to a maximum value during this period, and then decays when the stimulus disappears. For each item, VWM activity also increases towards an effective saturation limit, which is the maximum possible value divided by the number of items presented: here N=2, so the effective saturation limit is half the maximum possible value. When the target item is cued (black arrow; top) at a later time (yellow region), the non-target item(s) are removed from memory (grey trace), and activity associated with the target item (green trace) increases towards the maximum possible value. The level of activity (and hence the accuracy of memory recall) will vary more and more over time due to noise. VWM: visual working memory.

Image credit: Adapted from Figure 2 in the paper by Tomić and Bays, 2023.

The model constructed by Tomić and Bays can make specific testable predictions. For example, it predicts that if an item is cued for later recall while the sensory signal is still present, the working memory activity associated with the non-targets will decay rapidly, freeing up resources and thus increasing the working memory activity associated with the cued item. This leads to more accurate recall of the item. In contrast, if an item is cued for later recall once the sensory signal has approached zero, this ‘boost’ does not happen, and the item is not recalled as accurately. In addition, the working memory activity should increase with longer exposure to the stimulus and should decrease as the number of remembered items increases.

These predictions were confirmed through experiments with humans. Participants were shown visual stimuli while several factors were varied, including the number of items to be remembered, the duration of the stimulus, the time at which the item to be recalled was identified, and the time of the actual recall. The results of these experiments are consistent with the notion that, during recall, visual information is always read out from the same population of neurons.

The findings of Tomić and Bays are satisfying for their simplicity; what seemed to require two separate mechanisms is explained by a single framework aligned with many prior studies. However, models always require simplifications and shortcuts. For instance, much evidence indicates that both frontal lobe circuits and sensory areas contribute to the self-sustained maintenance of activity that underlies the short-term memory of sensory events (Pasternak and Greenlee, 2005). Therefore, visual working memory is likely the result of continuous recurrent dynamics across areas (DePasquale et al., 2018; Stroud et al., 2024). Furthermore, there is still debate about the degree to which visual working memory implies equal sharing of resources, as opposed to some items receiving larger or smaller shares (Ma et al., 2014; Pratte, 2018). Nevertheless, the proposed model is certainly an important advance that future studies can build upon.
